# Diagnostic Criteria in Attention Deficit Hyperactivity Disorder – Changes in DSM 5

**DOI:** 10.3389/fpsyt.2013.00049

**Published:** 2013-05-30

**Authors:** Sarah Steinau

**Affiliations:** ^1^Medical Faculty, University of FreiburgFreiburg, Germany

Attention deficit hyperactivity disorder (ADHD) is the most common neurobehavioral disorder in childhood, affecting large numbers of children throughout the world.

Because of the knowledge evolved from ADHD research, today's challenges are vast, including changes in terminology, fears of over-diagnosis, and over-medication of children.

What began as a case description, has now evolved in clinical trials, leading from observations of behavior to advances in neuroscience. The biggest challenge remains in the correct diagnosis of ADHD, though.

With a worldwide prevalence of approximately 5%, ADHD is very common (Polanczyk et al., 2007). ADHD in the United States even shows a prevalence among 8- to 15-years-olds of 8.7% and only a third of the ADHD patients have been treated consistently during the past year (Froehlich et al., 2007). In another age group (18- to 44-year-olds) the prevalence is about 4.4% (Kessler et al., 2006).

Male patients are six times more often diagnosed with ADHD in childhood than female patients and three times more often in the adolescence. The prevalence seems to be equivalent in all levels of IQ and socioeconomic status (Gaub and Carlson, 1997; Levy et al., 1997; Smalley et al., 2000; Pastor and Reuben, 2008).

The core symptoms of ADHD in DSM-IV-TR criteria (American Psychiatric Association, 2000) include inattention on the one hand and hyperactivity and impulsivity on the other hand, both having to be consistent to a degree that is maladaptive and inconsistent with developmental level, e.g., a 3-year-old's behavior is to be expected different from an 8-year-old's behavior.

Additional criteria include the chronicity of ADHD symptoms, meaning that there has to be a persistence of symptoms at least for 6 months, pervasiveness of ADHD symptoms (ADHD symptoms have to be present in different settings and locations, or at least in more than one location, e.g., family, community, workplace), some hyperactive-impulsive or inattentive symptoms must have been present before the age of 7 years and there must be clear evidence of interference with developmentally appropriate social, academic, or occupational functioning, e.g., you may have one of the symptoms, but if it is not impairing you or interfering with your functioning, a diagnosis cannot be made. At last but not least, the disturbance does not occur exclusively during the course of other serious mental disorders (e.g., pervasive developmental disorder/autism, schizophrenia, other psychotic disorder).

In the current DSM, there are three different ways of diagnosing ADHD. There is the combined type, e.g., both core symptoms are met for the past 6 months; there is the predominantly inattentive type and finally the predominantly hyperactive-impulsive type.

But what are the strengths of DSM-IV criteria? The committee of experts that has developed the DSM-IV criteria catalog clearly uses rigorous and empirically derived criteria, has looked at all rating scales for diagnosing ADHD and has reviewed the ADHD literature. Additionally, the impairment criterion has been given greater emphasis in the past few years.

Nevertheless there are certain weaknesses and controversies of DSM-IV criteria and it is important to have a second look: the age of onset criterion may not be justified (the age of 7 years does seem very restrictive), diagnostic item sets may be inappropriate for different developmental periods (e.g., not being able to sit quietly in a chair does not seem to be the right diagnostic item set for an adult, he does not need to listen or doesn't want to), diagnostic thresholds may not apply to older age groups (>16 years), there is no gender distinction in diagnostic thresholds and there is no lower age limit defined (<4 years).

So what are potential changes in DSM 5 criteria (American Psychiatric Association, 2013)? For a start, the age onset criterion shall be increased from 7 years of age to 12 years of age. Then there is the intent to contextualize and illustrate diagnostic item sets to fit lifespan (e.g., inattention in a child versus an adolescent versus an adult). Additionally, there are up to four new criteria for impulsivity (there have been only three dimensions compared to inattention or hyperactivity). And finally, the number of criteria needed for adolescents and adults is likely to be revised, for data has suggested two to three from all three dimensions (subtypes for inattention, hyperactivity, and impulsivity) would be best, which will probably increase the prevalence dramatically.
